# Online parent-targeted cognitive-behavioural therapy intervention to improve quality of life in families of young cancer survivors: study protocol for a randomised controlled trial

**DOI:** 10.1186/s13063-015-0681-6

**Published:** 2015-04-11

**Authors:** Claire E Wakefield, Ursula M Sansom-Daly, Brittany C McGill, Maria McCarthy, Afaf Girgis, Martha Grootenhuis, Belinda Barton, Pandora Patterson, Michael Osborn, Cherie Lowe, Antoinette Anazodo, Gordon Miles, Richard J Cohn

**Affiliations:** Kids Cancer Centre (KCC), Level 1, Sydney Children’s Hospital, High Street, Randwick, NSW 2031 Australia; Discipline of Paediatrics, School of Women’s and Children’s Health, UNSW Medicine, University of New South Wales, Level 3, Sydney Children’s Hospital, High Street, Randwick, NSW 2031 Australia; Sydney Youth Cancer Service, Prince of Wales/Sydney Children’s Hospital, High Street, Randwick, NSW 2031 Australia; The Royal Children’s Hospital Melbourne, Flemington Road, Parkville, VIC 3052 Australia; Murdoch Childrens Research Institute, Melbourne, Flemington Road, Parkville, 3052 Australia; Centre for Oncology Education and Research Translation (CONCERT), Ingham Institute for Applied Medical Research, South Western Sydney Clinical School, UNSW Medicine, The University of New South Wales, Campbell Street, Liverpool, NSW 2170 Australia; Pediatric Psychosocial Department G8-224, Academic Medical Center, Emma Kinderziekenhuis Meibergdreef 9, 1105 AZ Amsterdam, Netherlands; Children’s Hospital Education Research Institute, The Children’s Hospital at Westmead, Hawkesbury Road and Hainsworth Street, Westmead, NSW 2145 Australia; Discipline of Paediatrics and Child Health, Faculty of Medicine, University of Sydney, The Children’s Hospital at Westmead, Hawkesbury Road and Hainsworth Street, Westmead, NSW 2145 Australia; CanTeen, Level 11, 130 Elizabeth Street, Sydney, NSW 2000 Australia; Cancer Nursing Research Unit (CNRU), University of Sydney, Missenden Road, Camperdown, NSW 2050 Australia; Youth Cancer Service South Australia/Northern Territory, Royal Adelaide Hospital, North Terrace, Adelaide, SA 5000 Australia; Michael Rice Centre for Haematology and Oncology, Women’s and Children’s Hospital, King William Road, North Adelaide, SA 5006 Australia; Queensland Children’s Cancer Centre, Lady Cilento Children’s Hospital, Stanley Street, South, Brisbane, QLD 4101 Australia; Acute Services: Paediatric Consultation Liaison, Princess Margaret Hospital, Roberts Road, Subiaco, Perth, WA 6008 Australia

**Keywords:** Parent, carer, cancer, survivorship, intervention study, randomised controlled trial, psychological adaptation, quality of life, cognitive-behavioural therapy, Internet, E-health

## Abstract

**Background:**

Due to advances in multimodal therapies, most children survive cancer. In addition to the stresses of diagnosis and treatment, many families are now navigating the challenges of survivorship. Without sufficient support, the ongoing distress that parents experience after their child’s cancer treatment can negatively impact the quality of life and psychological wellbeing of all family members.

**Methods/Design:**

The ‘Cascade’ (*C*ope, *A*dapt, *S*urvive: Life after *CA*nc*E*r) study is a three-arm randomised controlled trial to evaluate the feasibility and efficacy of a new intervention to improve the quality of life of parents of young cancer survivors. Cascade will be compared to a peer-support group control and a 6-month waitlist control. Parents (n = 120) whose child (under 16 years of age) has completed cancer treatment in the past 1 to 12 months will be recruited from hospitals across Australia. Those randomised to receive Cascade will participate in four, weekly, 90-minute online group sessions led live by a psychologist. Cascade involves peer discussion on cognitive-behavioural coping skills, including behavioural activation, thought challenging, mindfulness and acceptance, communication and assertiveness skills training, problem-solving and goal-setting. Participants randomised to peer support will receive four, weekly, 90-minute, live, sessions of non-directive peer support. Participants will complete measures at baseline, directly post-intervention, one month post-intervention, and 6 months post-intervention. The primary outcome will be parents’ quality of life. Secondary outcomes include parent depression, anxiety, parenting self-agency, and the quality of life of children in the family. The child cancer survivor and all siblings aged 7 to 15 years will be invited to complete self-report quality of life measures covering physical, emotional, social and school-related domains.

**Discussion:**

This article reviews the empirical rationale for group-based, online cognitive-behavioural therapy in parents of children who have recently finished cancer treatment. The potential challenges of delivering skills-based programs online are highlighted. Cascade’s videoconferencing technology has the potential to address the geographic and psychological isolation of families after cancer treatment. Teaching parents coping skills as they resume their normal lives after their child’s cancer may see long-term benefits for the quality of life of the family as a whole.

**Trial registration:**

ACTRN12613000270718 (registered 6 March 2013).

**Electronic supplementary material:**

The online version of this article (doi:10.1186/s13063-015-0681-6) contains supplementary material, which is available to authorized users.

## Background

Although rare, cancer is a leading cause of death in children in developed countries [1]. After the shock of diagnosis, parents face the difficult dual challenges of supporting their child through debilitating treatment, whilst grappling with the possible death of their child [2]. Due to improved multimodal therapies, most children survive cancer [3]. It is often only when treatment ends that parents process the experience, at the very time when hospital-based psychosocial support is diminished [4]. Despite the acknowledged positive aspects of treatment completion [5,6], this is a vulnerable time for some parents, who can experience worsening quality of life (QoL), anxiety, depression, and feelings of helplessness [7,8]. Parents living in rural/remote areas appear most at risk of these poor outcomes [9]. Major themes of difficulty in the post-treatment period can include fear of cancer recurrence, isolation, and loneliness, with substantial unmet needs for information about relapse surveillance and how to ‘return to normality’ [10-12].

In the face of cancer, parents may lack the coping skills needed to manage the demands of their child’s treatment and survivorship, and others can develop maladaptive coping strategies under pressure [13]. Parental psychological adjustment problems may jeopardise their capacity to provide the ‘secure base’ that children need in times of stress [14] and can lead to less effective parenting [15,16]. Even after their child has been cured, distressed parents may express more anger toward their surviving child [17], listen less to their children [18], and have more negative parent–child interactions [19]. These parenting approaches may result in more behaviour problems [15] and distress [20,21] in young cancer survivors and their siblings [22]. The impact of poor parent coping may extend for years, with evidence that even 10 to 15 years post-diagnosis, coping in child cancer survivors may still be related to their mother’s coping [23]. Best-practice mental health interventions for children with cancer therefore need to target the family, not just the patient [24].

Evidence-based psychological interventions have the potential to reduce parental mental health burden in the ‘coming off treatment’ phase, thus curtailing longer-term difficulties. Best-practice interventions target modifiable processes associated with parents’ poor adaptation to their child’s cancer. For example, parents of children with cancer can use more ruminative thinking and defensive coping strategies [13], both of which can create conflict and poor family cohesion [25]. Interventions that increase parents’ use of adaptive coping strategies in the face of their child’s cancer may reduce their risk of depression and anxiety [26] and enhance their parenting skills [17]. Improving parents’ communication skills can also promote adaptive functioning in children with cancer [27]. Parents’ capacity to proactively solve problems and seek help when needed is also modifiable, yielding improved outcomes for children with cancer when targeted [13,28].

Skills-based interventions can be effective in parents of children with cancer, yielding medium-large effects [29]. However, few interventions have been rigorously evaluated. Most are also implemented face-to-face, limiting benefits to rural and remote families. It is clear that online cognitive-behavioural therapy (CBT) programs can be effective, with meta-analyses reporting medium-large effects for anxiety [30] and depression [31]. Trans-diagnostic programs that target co-morbid anxiety and depression may also be effective when delivered online [32]. Online programs also have the potential to reduce distress and improve wellbeing in those who care for someone with a medical condition [33]. Furthermore, group-based support is cost-effective and provides a unique context in which individuals can provide each other with emotional support, reflect on the commonalities of their experiences, and share resources [34]. Given that no efficacious, online programs currently exist to provide support for Australian parents at this recognised critical adjustment period [35], we developed a tailored intervention, ‘Cascade’, to meet their needs.

## Methods/Design

The Cascade study is a multi-site, randomised controlled trial (RCT) to assess the feasibility and efficacy of a new, online, CBT-based intervention for the parents or legal guardians (hereafter referred to as ‘parents’) of young cancer survivors (see Additional file 1 for a list of participating centres and ethical bodies). Parents of children under 16 years of age who have completed cancer treatment with curative intent and achieved remission in the past 12 months will be recruited and randomised to one of three arms: i) Cascade, ii) a peer-support group (PSG) control, or iii) a 6-month waitlist control. Informed consent will be obtained from all participants. The 1- to 12-month timeframe was chosen to maximise the benefit to participants, by delivering the coping skills intervention early in their child’s survivorship period. The intervention, named ‘CASCAdE’ (*C*ope, *A*dapt, *S*urvive: Life after *CA*nc*E*r), is delivered live, in real-time, to groups of 3 to 5 parents by a psychologist (hereafter referred to as ‘facilitator’) in four, weekly online sessions. Participants also receive an online introductory session with the facilitator before the group commences and a ‘booster’ session one month after the end of the group to facilitate consolidation of skills.

Cascade will be compared with the active PSG control in order to assess the relative benefits of peer-based support and contact with any additional psychological benefits gained from learning structured, skills-based coping strategies. ‘Peer-support’ type models of support are ubiquitous in community settings, yet few PSGs have been rigorously evaluated [36]. Consequently, little evidence exists to support the appropriateness of PSGs alone in addressing distress in parents of children with cancer. The PSG arm holds constant the amount of treatment contact, human interaction variables (such as facilitator warmth and interaction between participants), as well as controlling for participants’ expectations of receiving some form of treatment [37].

This study employs a three (treatment condition) by four (assessment point) factorial design. All participants will complete an assessment battery at baseline (T1: recruitment), immediately after participation in Cascade or the PSG control (T2), 5-weeks post-intervention/1-week post-booster session (T3), and 6-months post-intervention (T4). The waitlist group will be assessed at the same time points. After the 6-month waitlist period, waitlisted parents will be re-randomised to either Cascade or the PSG. They will complete a final, additional, assessment battery immediately after their group program (T5). All the children ages 7 to 15 years of participating parents (including the child cancer survivor and any siblings) will be invited to complete measures of depression, anxiety and peer relationships at the same time points as their parents (see Table 1).

**Table 1:** **Assessment schedule for the cascade study**

**Measure**	**Intake**	**Q1** ^**a**^	**During intervention** ^**b**^	**Q2** ^**c**^	**Q3** ^**d**^	**Q4** ^**e**^	**Q5** ^**f**^
Psychosocial Adjustment to Illness Scale-Interview form (PAIS)	**X**	**-**	**-**	**-**	**-**	**-**	**-**
Demographic data	**-**	**X**	**-**	**-**	**-**	**-**	**-**
Treatment Intensity Scale	**-**	**X**	**-**	**-**	**-**	**-**	**-**
Medical and general functioning^g^	**-**	**X**	**-**	**X**	**X**	**X**	**X**
Paediatric Quality of Life Inventory (PedsQL) Generic Core Scale^h^	**-**	**X**	**-**	**X**	**X**	**X**	**X**
Paediatric Quality of Life Inventory (Family Impact Module)	**-**	**X**	**-**	**X**	**X**	**X**	**X**
EQ-5D-5 L	**-**	**X**	**-**	**X**	**X**	**X**	**X**
Parenting Self Agency Measure (Revised)	**-**	**X**	**-**	**X**	**X**	**X**	**X**
CBT skills use	**-**	**X**	**-**	**X**	**X**	**X**	**X**
PROMIS parent mental health and functioning short-form items^i^	**-**	**X**	**-**	**X**	**X**	**X**	**X**
California Psychotherapy Alliance Scale - Group (CALPAS-G)	**-**	**-**	**-**	**X**	**-**	**-**	**X**
Intervention satisfaction items	**-**	**-**	**-**	**X**	**-**	**-**	**X**
Emotion Thermometers Tool	**X**	**-**	**X**	**-**	**-**	**-**	**-**
Homework Compliance Scale	**-**	**-**	**X**	**-**	**-**	**-**	**-**
Working Alliance Inventory - Short	**-**	**-**	**X**	**-**	**-**	**-**	**-**

^**a**^Q1 = Baseline; ^**b**^During intervention = weekly prior to intervention sessions 2 to 4; ^**c**^Q2 = post-intervention.

^**d**^Q3 = Week 5 follow-up, ^e^Q4 = 6 month follow-up; ^f^Q5 = after waitlist participants have completed the intervention.

^g^Including other psychological support received; ^h^Parent proxy and child self-report; ^i^Items assess parent depression and anxiety symptoms.

### Aims and hypotheses

This RCT aims to assess the following:

1. The feasibility of implementing Cascade nationwide, including the recruitment procedure, response/attrition rates and cost.
2. The efficacy of Cascade in improving the QoL of parents (the primary outcome). Secondary outcomes include parent depression, anxiety, and parenting self-agency, as well as social and emotional functioning of the children in the family.

We hypothesise that delivering Cascade will be feasible and acceptable. We also hypothesise the following:

1. Both the PSG and Cascade participants will show greater improvements in QoL compared with the waitlist control, measured from T1 to T2.
2. Participants who complete Cascade will show greater improvements in their QoL compared to participants who complete the PSG, measured from T1 to T2.

### Participants

This study will recruit 120 parents (approximately 40 in each arm). This sample size will allow medium-to-large differences in parent QoL to be detected with a power of 80% at a two-tailed significance level of 0.05 (assuming Cohen’s d = 0.65 as the difference in change from time 1 to time 2 for any pair of groups, standardised on the pooled within-group standard deviation). This effect size is clinically significant [38]. It is anticipated that approximately 375 parents will need to be approached to achieve a final sample of 120 participants (assuming a 40% response rate and 20% attrition rate).

#### Inclusion criteria

Eligible parents will meet the following inclusion criteria: i) have a child under 16 years of age who has completed cancer treatment with curative intent in the past 12 months; ii) be able to give informed consent; iii) be able to read English; iv) be able to provide the contact details of a trusted health professional, such as their local general practitioner; and v) be able to access the Internet in a private location (see also *Access considerations* below). Children will be eligible if they are aged 7 to 15 years and, in the opinion of the parent, are capable of reading at a Grade Two level. In this study, we are interested in parents of young cancer survivors aged less than 16 years, and so we matched siblings to this age range. Child participants will require parental consent.

#### Exclusion criteria

Parents will be excluded if, during the initial intake interview, they i) have insufficient English language skills to complete the interview; ii) demonstrate very high levels of distress, anxiety, and/or depression on the Emotion Thermometers Tool (that is, scores ≥ 7) [39] and endorse serious suicidal intent; iii) endorse symptoms of psychosis or substance abuse; or iv) have a child who is currently on active treatment, has relapsed, or is in palliative care. Any participant who is excluded will be provided with appropriate referral options, if desired.

#### Access considerations

To participate in the study internet access and a suitable computer set-up is required. This includes access to a computer/tablet that has a microphone and web-camera and can be used privately and uninterrupted once per week for four weeks. Participants will be loaned an insured tablet and/or web-camera and internet access USB device if needed (the costs of which are covered by Cascade’s project grant).

#### Participant recruitment

Potential parent participants will be mailed an invitation package comprising a personalised invitation letter from the Head of Oncology or their child’s treating oncologist at their child’s treating centre, a consent form, and an opt-in card, as well as a separate child assent form. The research officer will contact all parents who opt in to assess their technology needs and, if a loan is required, request that participants sign and return a written contract agreeing to use the equipment solely for study purposes.

**Figure 1:**
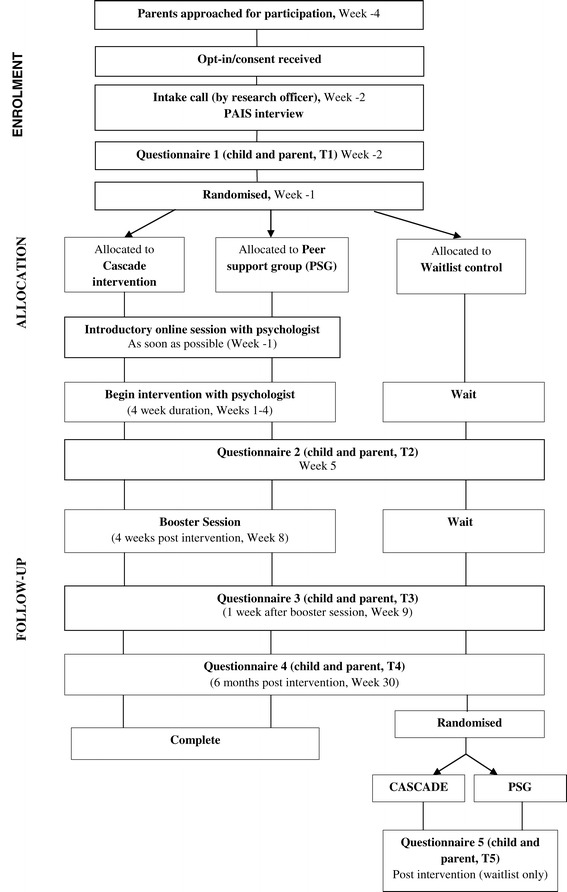
Cascade study flowchart.

Recruitment will occur in 5 × 12 week blocks, such that approximately 75 parents will be invited 4 weeks prior to Week 1 of each block. Using our expected response and attrition rates, it is envisaged that five iterations (that is, 5 × 12 week blocks), each attracting approximately 30 parent opt-ins, will be required to achieve the target sample. This means that five mail outs (approximately 75 parents at a time) will occur during 2014/5. All fully consented participants will be telephoned by the research officer 2 weeks prior to Week 1 to administer the Psychosocial Adjustment to Illness Scale Interview (PAIS) [40]. Participants will also complete the first online questionnaire at this time. See Figure 1 for the study flowchart.

### Randomisation

Participants will be randomised to one of the three arms using a flexible biased urn method of randomisation, which adapts to the degree of imbalance between groups in a dynamic manner over the trial [41]. This method is superior to standard stratification in balancing groups across multiple covariates [42,43], whilst also being a suitable method when groups remain small [41,44,45]. The groups will be balanced across two factors: i) severity of distress as measured by the Emotion Thermometers Tool [39] and ii) degree of rural/remoteness as assessed by the Accessibility/Remoteness Index of Australia [46]. An independent researcher will electronically randomise participants to treatment group.

### Interventions

#### Cascade

Cascade is guided by the family systems illness model [47]. This evidence-based framework conceptualises resilience in families as a multifaceted process involving the interaction between negative sequelae (stressors) and positive responses (coping strategies), with a key role for family members’ appraisal of the stressor in determining adaptive coping responses. Cascade derives its proposed core mechanisms of change from CBT and addresses both positive and negative outcomes after cancer. CBT may be particularly effective in improving QoL in carers of cancer patients if it targets communication and problem solving skills (both addressed in Cascade) [48]. Each Cascade module applies CBT techniques to the key domains of concern identified in our previous research [4,49] (see Table 2). Our strengths-based approach recognises that families are resilient and competent [31], which means that Cascade builds on psychological strengths while mitigating negative symptoms in the broadest possible group.

**Table 2:** **Cascade intervention content**

	**Module title and description**	**Relevant cognitive-behavioural therapy strategies**
**Module 1**	*‘What just happened to us?!’*	Peer discussion to normalise range of typical parent experiences.
	*Processing the cancer experience and getting back to some form of ‘normal’.*	Behavioural activation to improve mood, fatigue, and activity levels.
**Module 2**	*‘How has cancer changed the way I think?’*	Peer discussion to normalise increase in frequency and intensity of worries after cancer.
	*Managing upsetting thoughts and worries about cancer and everything else.*	Psycho-education about common unhelpful thinking styles
		Cognitive challenging.
**Module 3**	*‘Out of your head and back into life’*	Peer discussion to normalise existential concerns and highly distressing worries (for example, about child’s possible death).
	*Mindfulness and acceptance-based strategies for easing the struggle with ‘bigger’ existential concerns and emotional pain.*	Practical problem solving strategies for problems with logical solution(s).
		Cognitive strategies for disengaging from patterns of unhelpful thinking (for example, attention training, mindfulness exercises).
**Module 4**	*‘Looking forward’*	Peer discussion to normalise changes to relationships and feelings of isolation after the cancer experience.
	*Skills for fostering meaningful relationships, accessing social support and living a rich and fulfilling life after cancer.*	Supportive discussion and problem-solving strategies to stimulate support seeking from family and network.
		Assertive, effective communication skills strategies.
**Booster**	*Individual ‘catch-up’ session with each parent one month after the end of the group.*	Assist participant to identify challenging situations in the past month according to cognitive-behavioural model, and review helpful coping skills.
		Review goal from the start of the program - discuss what has helped and what remains challenging in this area.

Participants randomised to Cascade will participate in four, live, weekly, 90-minute sessions facilitated by a psychologist. Each group will comprise a psychologist and three to five participants with mixed cancer experiences (such as the age of their child and their child’s type of cancer). Participants also receive a purposely developed workbook, outlining the content of each session, providing additional examples and suggesting ‘homework’ activities to facilitate skill development. Additional files 2, 3 and 4 show details of the workbook content and graphic design.

Sessions will be delivered through WebEx (Cisco WebEx, USA). WebEx requires a computer with standard browser, a high-speed internet connection, and a webcam. WebEx is a secure, password-protected video-conferencing program that allows up to six participants to be seen on the screen simultaneously, similar to group Skype™. Participants will receive a reminder text message on their cellular phone 24 hours before their session, which will also serve as their reminder to complete the Emotion Thermometers Tool [39] and homework compliance scale (see *Assessments*).

#### Peer-support group (active control)

The PSG control is delivered in an identical manner to Cascade (via WebEx, up to five participants per group), with the same frequency of contact (four weekly 90-minute sessions) and availability of peer-based group discussion. The PSG is delivered by the same facilitator as Cascade (see *Treatment fidelity* for further discussion). Like Cascade, it also involves supportive counselling to normalise the range of parent experiences and provides parents an opportunity to give and receive emotional/practical support. During each session, parents are encouraged to exchange information about a nominated topic (matched to those addressed in Cascade for example, ‘relationships and social support’). The key distinction between Cascade and the PSG is that the PSG does not include directive, structured teaching of specific, CBT-based coping skills. The PSG in this trial will adhere to best practice guidelines [50] and is manualised to ensure standardisation across all sessions.

### Procedures

Following recruitment, participants will complete a telephone intake interview with a Cascade research officer to further screen for participant eligibility, orient participants to the study procedures, ascertain any technical needs, and complete the PAIS [40].

Participants will then be randomly allocated to a study arm. During Weeks 1 to 4 of each 12-week block, those allocated to Cascade and the PSG will participate in their allocated intervention. Waitlist controls will be assessed at the same time points as the intervention groups, and will be randomly allocated to either Cascade or PSG during weeks 26 to 30. The waitlist group will also complete a final questionnaire (Q5) after participating in the intervention. Parents will be sent links via email to complete the questionnaires (see *Data management and analysis*) and will be encouraged to supervise their child/ren while they complete the questionnaires, if desired.

#### Study integrity

This study is listed on the Australian New Zealand Clinical Trials Registry ACTRN12613000270718, and has undergone rigorous multidisciplinary peer and consumer review. It is endorsed by the Australian New Zealand Children’s Haematology Oncology Group (ANZCHOG).

Ethical approval has been obtained from Sydney Children’s Hospital, Children’s Hospital at Westmead, Royal Children’s Hospital Melbourne, Monash Children’s Hospital Melbourne, Queensland Children’s Cancer Centre, Women’s and Children’s Hospital Adelaide, and the Royal Adelaide Hospital. This study complies with the CONSORT guidelines [42] by using the following: a) standardised assessment measures; b) blind assessments; c) standardised assessor training and inter-rater reliability checks; d) manualised, replicable procedures for all conditions; e) random allocation; and f) treatment fidelity checks.

#### Treatment fidelity

Both treatment groups will be facilitated by the same person to prevent confounds (for example, attributes such as age, sex and communication style), each of which could impact group retention/efficacy. Any variation or systematic biases between groups will be detected and corrected by the independent assessors during treatment fidelity checks of a random 15% of all video-recorded sessions (the validated ‘Method of Assessing Treatment Delivery’ advises a minimum of 11%) [51]. All pre- and post-treatment outcome measures will be administered by the research officer, who will be blind to group allocation. In compliance with the CONSORT guidelines, the research officer will report on which condition they believe each participant was in at the end of the study.

To ascertain why this intervention may not be tolerated by all parents, exit interviews will be collected for all who leave the study prematurely, as well as for 15% of those who complete the intervention to collect in-depth data on participants’ likes and dislikes and to solicit ideas for improvement.

#### Safety monitoring

**Figure 2:**
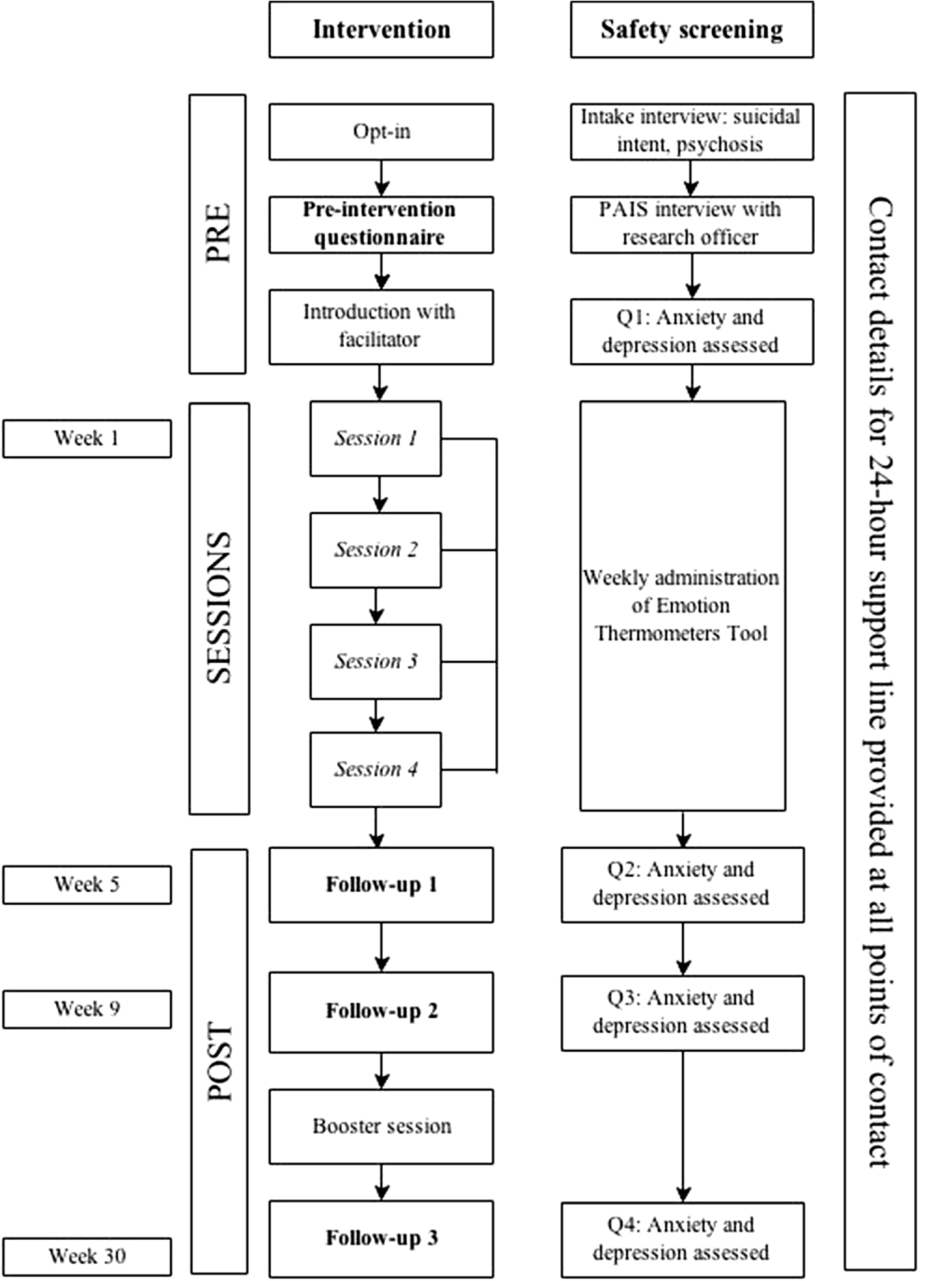
Cascade safety monitoring procedures.

This trial includes safety monitoring and management procedures at multiple project stages (see Figure 2). The intake interview carefully screens for acutely suicidal/severely depressed participants. Participants will also be regularly screened for mood deterioration during intervention participation when they complete the weekly Emotion Thermometers Tool [39] (see *Assessments*). Any deterioration in mood of more than three points on the tool will trigger protocols involving the facilitator contacting the participant to discuss their emotional state and a meeting between the researchers to develop a management plan (which may include contacting their nominated health professional, with the participant’s consent).

### Assessments

#### Feasibility

The feasibility of Cascade will be determined by recording the (i) time taken to recruit sufficient participant numbers, (ii) proportion of participants who required loaned technology, (iii) time taken to complete and return questionnaires, (iv) study response rate (feasible at 40%), (v) attrition rates of each arm (feasible at 20%), and (vi) proportion of children who participate. Feasibility will also be assessed by examining the flow-through of the study (from opt-in to final questionnaire completion), using medians and ranges.

#### Demographic measures

Table 1 summarises the planned assessments. Information on parent age, sex, education, employment status, and family structure will be collected, as well as information on the child’s diagnosis and treatment regimen using standardised items adapted from the Childhood Cancer Survivor Study [52]. Information such as whether the child relapsed or received a bone marrow transplant will be obtained. This data will be sufficient for a qualified paediatric oncologist to grade the child’s treatment intensity according to the validated Treatment Intensity Scale [53] will be included in order to assess the severity/intensity of the child survivor’s medical treatment. Information on the gender and age of all child participants will be obtained. Parent participants will also be asked to report on all forms of psychological support accessed at each time point.

#### Parent/caregiver psychosocial functioning

There is little consensus regarding the relative superiority of generic versus disease-specific psychosocial measures in psycho-oncology. Disease-specific measures often appeal to researchers due to the difficulty ascertaining ‘clinical’ change in populations that may only be distressed at sub-clinical levels [54,55]. However, generic psychological measures facilitate comparison to healthy norms, which can be clinically and empirically advantageous [56]. As such, the Cascade trial uses both generic and disease-specific indicators of psychosocial functioning.

The Pediatric Quality of Life inventory (PedsQL) Family Impact Module [57] was selected as the primary psychosocial outcome variable as it assesses parent/caregiver functional concerns specifically related to their child’s illness. Four purposefully developed and pilot-tested items were also added to specifically index parents’ concerns (if any) about their child’s cancer recurring. The widely-used EQ-5D-5 L [58] is a generic measure that will assess parents’ wellbeing in five domains: mobility, self-care, engagement in usual activities, physical pain/discomfort and anxiety/depression.

The Psychosocial Adjustment to Illness Scale-Interview Form (PAIS) [40] will be administered over the telephone. The PAIS assesses adjustment of patients and parents/carers to illness across seven domains: health care orientation, vocational environment, domestic environment, sexual relationships, extended family relationships, social environment and psychological distress.

The Parent Self-Agency Measure (Revised) [59] provides an index of parents’ general confidence in their parenting behaviours. Parenting competence is of interest in Cascade due to its bi-directional relationship with child development and adjustment outcomes. Importantly, parents’ confidence in their ability to parent and associated positive parenting practices may be a protective factor for families under stress [60].

Finally, items from the Patient-Reported Outcomes Measurement Information System (PROMIS) short-form questionnaires will be used to assess depression and anxiety symptomology. PROMIS has excellent psychometric properties superior to many other measures available to assess emotional functioning [61].

#### Child psychosocial functioning

The Paediatric Quality of Life Inventory (PedsQL) Generic Core [62] parent proxy-report scale will be used to assess the cancer survivor’s quality of life in physical, emotional, social, and school-related domains of functioning. In a recent review of QoL measures for children, the PedsQL emerged as a feasible and valid tool that is widely used in cancer and chronic disease [63]. All participating children (the child survivor and/or any siblings) will also complete a child self-report version of the scale.

#### Intervention engagement and impact

To assess participants’ mood in a more dynamic manner across the intervention, parents will complete the Emotion Thermometers Tool [39] each week, 24-hours prior to participating in their weekly session (Cascade or PSG). At the same time, parents will complete the 6-point Homework Compliance Scale [64] to assess compliance with the home practice exercises. CBT skills used will also be assessed using 10 purposely developed and pilot-tested items; for example, assessing participants’ ability to ‘recognise unhelpful thoughts and how they are making me feel’ (response options range from ‘not at all’ to ‘a lot’). The Working Alliance Inventory - Short (WAI- S) [65] and The California Psychotherapy Alliance Scale - Group (CALPAS-G) [66] will be used to assess participants’ perceptions of the therapeutic working alliance, during and after the intervention.

#### Satisfaction with intervention

After participating in Cascade or the PSG, parents will provide ratings of specific intervention elements to determine their acceptability. Open-ended questions will also be used to elicit views about the benefit and/or burden of participating and suggestions for improvement.

### Data management and analysis

All measures (excluding the PAIS telephone interview) will be administered online through Key Survey (WorldAPP, Braintree, MA, USA) at all time points, unless paper versions are requested by participants. Key Survey enables participants’ data to be securely downloaded to files amenable to statistical analysis using the Statistical Package for the Social Sciences, version 18.0 (SPSS, Inc., Chicago, IL, USA).

#### Statistical analyses

This trial will employ ‘intention-to-treat’ and ‘as-treated’ analyses. Analyses will be based on mixed random-intercept models that will assess differences between the groups in terms of change in QoL from T1 to T2 (the primary analysis), from T1 to T3, and from T1 to T4. Random intercept models, which utilise maximum-likelihood estimation, provide more efficient estimates of effects with unbalanced data than the traditional repeated measures approach [67]. Multiple regression analyses will be conducted using T1 data to identify demographic and other factors that contribute to treatment outcome. Multiple comparisons will be used to test a priori hypotheses and to conduct post-hoc testing, with the alpha rate adjusted using the Holm-Bonferroni method [68].

## Discussion

This paper outlines the protocol for a multisite trial of a novel online intervention for parents of children who have recently completed treatment for cancer, entitled Cascade. The Cascade program is unique because (i) it targets parents *and* assesses the possible impact of the intervention on children in the family and (ii) its online delivery reduces geographical and physical isolation. The planned RCT is methodologically rigorous since it follows gold-standard guidelines, includes both an active and waitlist control, and employs strict treatment fidelity assessments.

The technology used to deliver Cascade is an important innovation, as it enables the provision of evidence-based support to families dispersed across metropolitan, rural and remote regions. Cascade is part of a broader telehealth movement bringing about change in the way that mental health care is delivered worldwide, in response to factors such as geographical isolation and limitations of time and resources [69-72]. There is growing evidence supporting the potential of telepsychology in treating disorders such as anxiety and depression [31,73,74]. Evidence suggests that core aspects of CBT such as cognitive challenging, role-playing and modelling, setting up behavioural experiments and homework assignments, translate well over videoconferencing [75]. Further, videoconferencing does not appear to diminish facilitator competence, adherence, or patient perceptions of rapport or empathy conveyed by the facilitator [74]. However, as most ‘online therapies’ are self-guided by the user with telephone/email-based support [76], few manualised treatment programs or best-practice guidelines exist to guide Cascade in aspects relating to therapy process and online interaction. This may mean that facilitator practice effects occur across the study period, as the facilitator gains competency in anticipating, and managing, challenges in the videoconferencing environment.

This study is strengthened by the inclusion of both an active and a waitlist control group. The waitlist group controls for the possibilities that parent distress may dissipate in the first weeks after treatment completion and/or that clinical services may change or improve over the recruitment period. However, the additional use of a non-specific treatment arm (the PSG) is now considered gold standard. Active controls better manage participants’ expectations of receiving some form of treatment. This trial therefore enables an assessment of whether an intensive, structured, skills-based intervention such as Cascade confers any benefit over peer support alone. This question has important clinical, as well as economic considerations, as the two strategies have different implications for resources, training and time commitments required.

The planned treatment fidelity assessments will enable an examination of the relative benefits of a structured, CBT-based intervention when compared with a non-directive, peer-support group model. It is possible and likely that although the facilitator does not teach the same structured, CBT-based coping skills in the PSG, the peer groups may nevertheless spontaneously discuss adaptive coping skills, unhelpful thinking styles, or stress reduction strategies. The treatment fidelity assessment will allow an examination of the extent to which such skills-based discussion is facilitated, or directed, by the Cascade facilitator, and the proportion of session time spent discussing adaptive coping strategies. By conducting fidelity checks concurrently across the trial any significant content overlap initiated by the facilitator will be able to be corrected. This process will ensure that despite some likely overlap in content, it will still be possible to distinguish between the two arms in terms of mechanisms of change. This is critical in order to make recommendations for future intervention design.

Despite its strengths, the Cascade study design also has methodological vulnerabilities. The three-armed design will increase the time it takes to recruit sufficient participant numbers. In addition, the 6-month follow-up is another aspect of the study design that, although methodologically important, may add complexities to final data analyses. Participants may differ in terms of psychosocial support services they receive in this time, and the number of other parents of childhood cancer survivors they come into contact with. Individual differences in additional support services and peer support is likely to be important throughout the trial. These factors will require careful monitoring and documentation, and will need to be taken into account in data analyses/interpretation.

In sum, Cascade is a selective preventative program with the potential to avoid mental health problems in parents and other family members by equipping parents with coping skills to manage the challenges of the survivorship period. This study trials a new model of healthcare delivery that can extend the reach of support to isolated populations worldwide. If this study demonstrates significant improvements in QoL, Cascade will be made available for use with the parents of childhood cancer survivors across Australia, with the potential to be delivered internationally as well.

## Trial status

This is a clinical trial with ongoing patient recruitment. Recruitment for this project commenced in September 2014 and is expected to be completed by June 2016. This trial is recorded under the number ACTRN12613000270718.

## Additional files

Additional file 1:
**Cascade participating centres and ethical bodies.**
Additional file 2:
**Cascade workbook graphics – Title page.**
Additional file 3:
**Cascade workbook graphics – Parent quotes.**
Additional file 4:
**Cascade workbook graphics – Psychoeducation.**

## Abbreviations

CBT: cognitive-behavioural therapy
ETT: Emotion Thermometers Tool
PAIS: Psychosocial Adjustment to Illness Scale
PROMIS: Patient-reported Outcomes Measurement Information System
PSG: peer support group
QoL: quality of life
RCT: randomised controlled trial
SPSS: Statistical Package for the Social Sciences
