# Supplementary material for: Online parent-targeted cognitive-behavioural therapy intervention to improve quality of life in families of young cancer survivors: study protocol for a randomised controlled trial
Source: Trials. 2015 Apr 11;16:153. doi: 10.1186/s13063-015-0681-6 (PMC4395969; doi:10.1186/s13063-015-0681-6)
Supplement: Additional file 1: — Cascade participating centres and ethical bodies. [file 13063_2015_681_MOESM1_ESM.pdf]

Hunter New England Human Research Ethics Committee
(*Under the Australian National Mutual Acceptance Scheme*)

HREC/14/HNE/44

Women's and Children's Hospital, Adelaide<br><br>Women's and Children's Hospital Network<br><br>SSA/14/WCHN/60	Royal Children's Hospital, Brisbane<br><br>Children's Health Queensland Hospital and Health Service<br><br>SSA/14/QRCH/95
The Children's Hospital at Westmead<br><br>The Sydney Children's Hospital Network<br><br>SSA/14/SCHN/219	The Sydney Children's Hospital<br><br>The Sydney Children's Hospital Network<br><br>SSA/14/SCHN/156
