# Supplementary figures and images for: Online parent-targeted cognitive-behavioural therapy intervention to improve quality of life in families of young cancer survivors: study protocol for a randomised controlled trial
Source: Trials. 2015 Apr 11;16:153. doi: 10.1186/s13063-015-0681-6 (PMC4395969; doi:10.1186/s13063-015-0681-6)

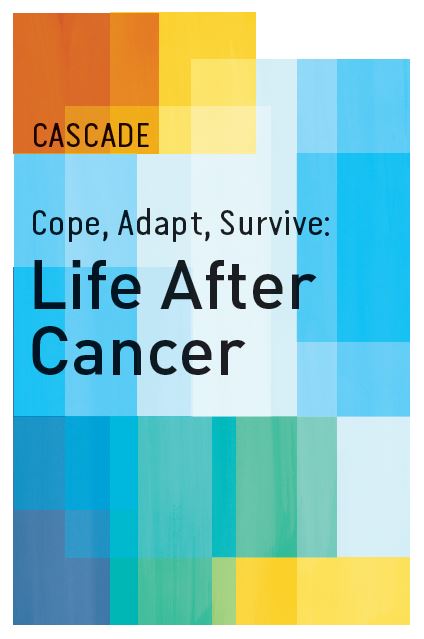

Supplement: Additional file 2: — Cascade workbook graphics – Title page. [file 13063_2015_681_MOESM2_ESM.jpeg]

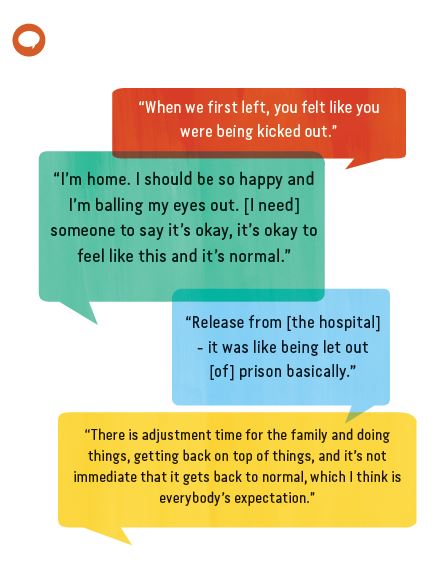

Supplement: Additional file 3: — Cascade workbook graphics – Parent quotes. [file 13063_2015_681_MOESM3_ESM.jpeg]

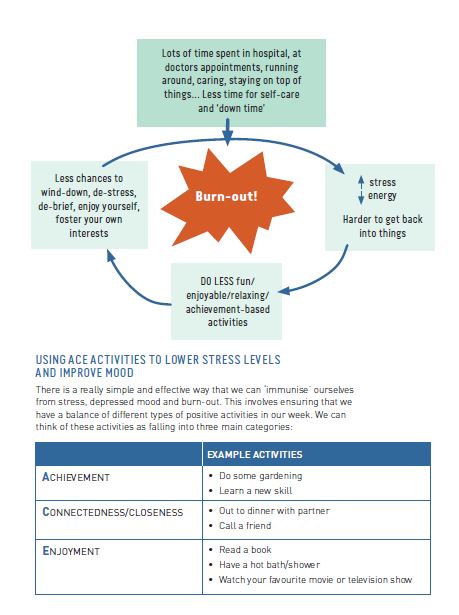

Supplement: Additional file 4: — Cascade workbook graphics – Psychoeducation. [file 13063_2015_681_MOESM4_ESM.jpeg]
